# Data on the thermal method of odour elimination implemented in the Polish agro-food consortium

**DOI:** 10.1016/j.dib.2021.106987

**Published:** 2021-03-22

**Authors:** Zygmunt Kowalski, Agnieszka Makara

**Affiliations:** a Mineral and Energy Economy Research Institute Polish Academy of Sciences, Wybickiego 7, 31-261 Kraków, Poland; b Faculty of Chemical Engineering and Technology, Cracow University of Technology, Warszawska 24, 31-155 Kraków, Poland

**Keywords:** Circular economy, Meat waste, Meat-bone meal, Recycling, Odour elimination, Thermal method

## Abstract

The data presented in this article are related to the research paper entitled “The circular economy model used in the Polish agro-food consortium: a case study” published in the Journal of Cleaner Production 284 (2021) 124751. The utilisation of meat waste for the production of meat-bone meal is the Farmutil's consortium main activity. The Oxidor system was developed for the combustion of all the odour emitted from meat-bone meal production. This improved the relationship of consortium with the public. Flow-sheet of the unit for thermal oxidation of odour, scheme and image of the thermo-oxidator were presented. The steam produced in the Oxidor system is recycled and re-used for meat-bone meal production. This is an example of new organisational solutions in circular economy originating from a physical flow concept in which energy flows are used as a result of closed-loop recycling.

## Specifications Table

**Table:** 

Subject	Management of Technology and Innovation
Specific subject area	The thermal method of odour elimination used in agro-food consortium
Type of data	Figures, Tables
How data were acquired	Data from the Oxidor system developed for combustion of all the odour from meat-bone meal production and effects of its implementation.
Data format	Analysed
Parameters for data collection	Farmutil's main activity is the utilisation of meat waste for the production of meat-bone meal (MBM). The Oxidor system developed for combustion of all the odour emitted from MBM production was analysed. The implemented thermal method eliminates odour emission from MBM production what was very important in terms of environment pollution and reduction of hazard for human health. This improved the relationship of Farmutil's consortium with the public.
Description of data collection	Following data were collected in Farmutil: flow-sheet of the unit for thermal oxidation of odour contained in vapours from meat-bone meal production and the air from production objects; scheme and image of the thermo-oxidator; description of Oxidor system and its operating parameters; measurement methods and pollutants concentration in exhaust gases after thermo-oxidator in relation to the standards.
Data source location	Institution: Farmutil HS Inc. City/Town/Region: Śmiłowo Country: Poland
Data accessibility	Data is provided in this article
Related research article	Z. Kowalski, A. Makara. The circular economy model used in the Polish agro-food consortium: a case study, J. Clean. Prod. 284 (2021) 124751 https://doi.org/10.1016/j.jclepro.2020.124751

## Value of the Data

• Farmutil's main activity is the utilisation of meat waste for the production of meat-bone meal (MBM). Main problem of MBM production was emission of odours.
• The Oxidor system has been used for combustion of all the gaseous fumes from technological MBM operations and air from production rooms, containing odour. Flow-sheet of the unit for thermal oxidation of odour contained in flue gases, scheme and image of the thermo-oxidator were presented.
• The steam produced in Oxidor system is recycled and re-used for MBM production (in-process heat recycling). This is an example of new organisational solutions in circular economy (CE) originating from a physical flow concept in which energy flows are used by Farmutil as a result of closed-loop recycling.
• Measurement methods and results of measurements of pollutants concentration in exhaust gases after thermo-oxidator in relation to the standards were presented.

## 1 Data Description

The production management system used in Farmutil agro-food consortium is an example of micro-level applications in industrial practice for the basic elements of the circular economy [1]. Farmutil's main activity is the utilisation of meat waste for the production of meat-bone meal (MBM). MBM units, due to the applied technology and apparatus solutions, can be considered as the most modern in the European Union (EU). Their actual capacity allowed utilising over 300,000 t of meat waste. One of the most serious problems of MBM production was the emission of odours from this production. We analysed the Oxidor system developed for combustion of all the odour emitted from MBM production. Methods of odour emission measurements were widely described in [2]. The implemented thermal method is highly effective, but expensive [3]. Its implementation eliminates odour emission from MBM production what was very important in terms of environment pollution and reduction of hazard for human health [4]. This improved the relationship of Farmutil consortium with the public [1].

The raw data are presented in the supplementary material

Supplementary Material

Table 1 contains the chemical composition of ashes after combustion of meat bone-meal.

Table 2 presents characteristics of exhaust gases after thermo-oxidator and gas parameters in the emitter channel.

Table 3 presents average concentrations of inorganic compounds NO_x_, CO, CO_2_, O_2_, SO_2_ in gases from the thermo-oxidator.

Metal concentrations in exhaust gases after thermos-oxidator are presented in Table 4.

Results of measurements of organic carbon (C_org_) in exhaust gases after thermo-oxidizer are shown in Table 5.

Measurement results of dioxins and furans concentrations in flue gas after thermo-oxidator. List of 2,3,7,8-PCDDs and PCDFs congeners and the corresponding TEF toxicity factors are presented in Table 6.

Table 7 presents results of measurements of pollutants concentration in exhaust gases after thermo-oxidator in relation to the Polish and EU standards.

## 2 Experimental Design, Materials and Methods

The Oxidor system has been used for combustion of all the gaseous fumes from individual technological MBM operations and air from production rooms, containing odour (total quantity - 200 million m^3^/y) and production of steam based on heat from post-combustion gases. Produced steam is recycled and used for MBM production (in-process recycling). This is an example of new organisational solutions in CE originating from a physical flow concept in which energy flows are used by Farmutil as a result of closed-loop recycling [1].

**Fig. 1:**
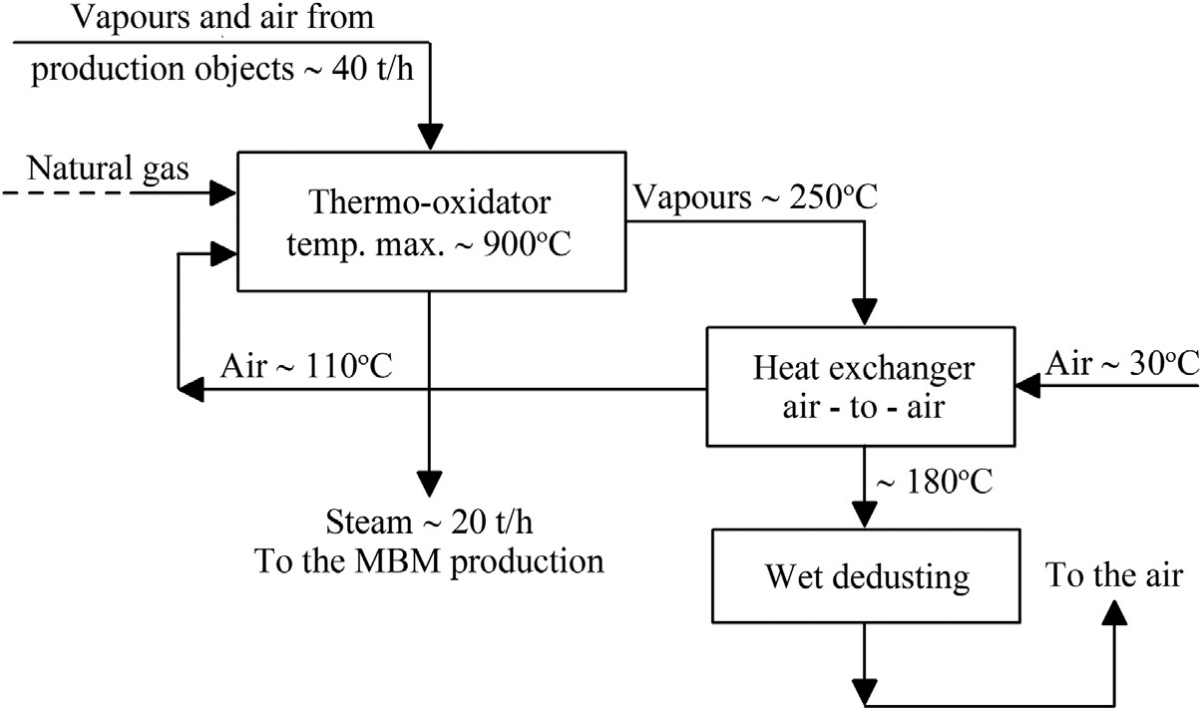
Oxidor system. Flow-sheet of the unit for thermal oxidation of odour contained in vapours from meat-bone meal production and the air from production objects.

**Fig. 2:**
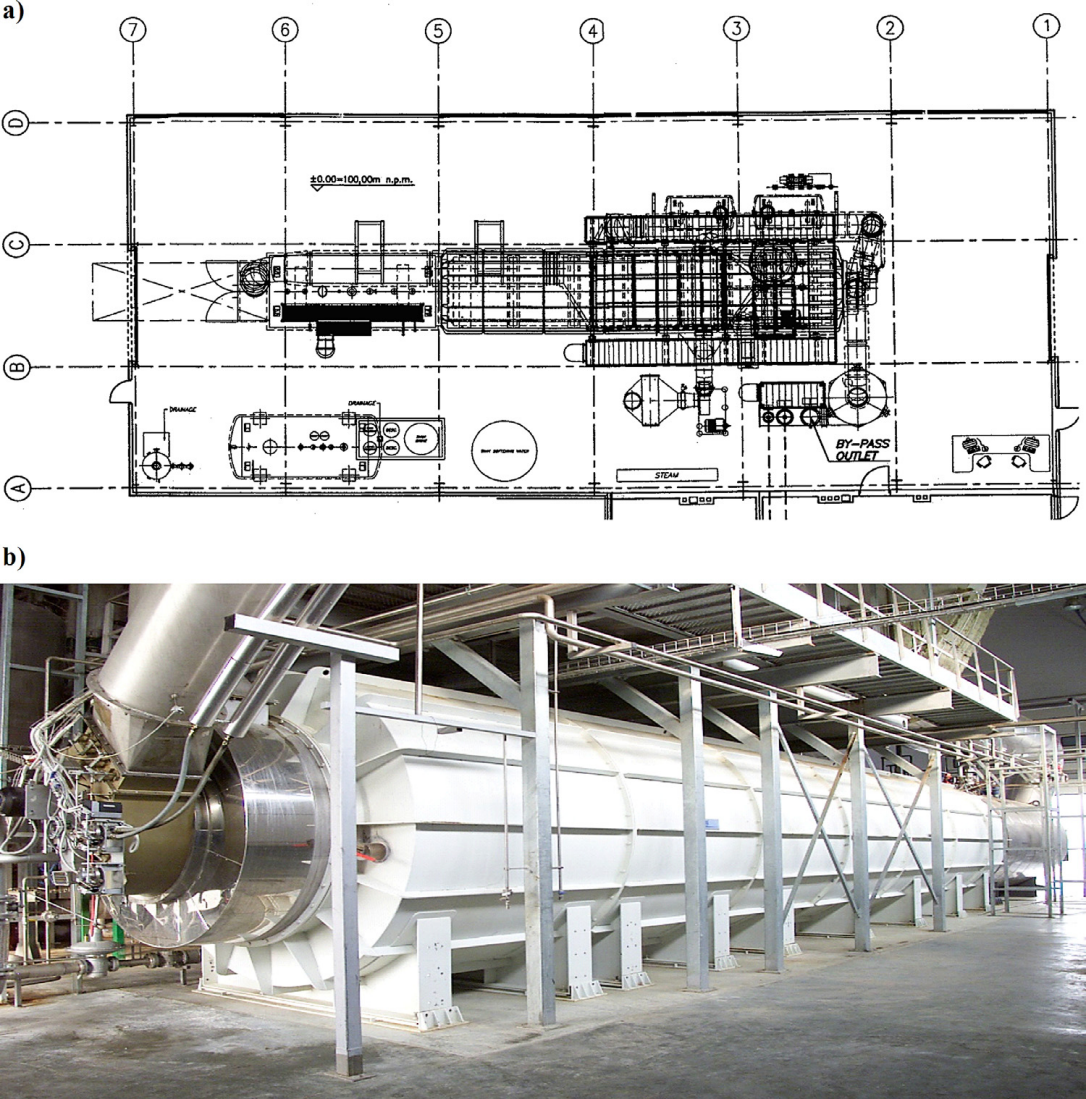
Scheme (a) and image (b) of the thermo-oxidator.

Flow-sheet of the unit for thermal oxidation of odour contained in vapours from meat-bone meal production and the air from production objects is presented in Fig. 1. Scheme and image of the thermo-oxidator are shown in Fig. 2.

### 2.1 Oxidor system – operation description

Vapours from MBM production were treated and discharged through cyclones and a pipeline system to the T01C oxidation chamber equipped with T01A/B rotary burners through the T03 vapour line and process air from the production hall through the T02 air line. Vapours and air will be introduced to the T01A/B burners, which will eliminate the need to suck in cold air from the outside in order to ensure excess air necessary for the proper functioning of the burners. Prior to combustion, the process air will be preheated in the “air-to-air” heat exchanger T01D. The air that gives off heat in the exchanger will come from the outlet of the T01F steam boiler. The process of oxidation of organic pollutants causing unpleasant doors will be carried out for minimum 2.02 s.

### 2.2 Basic data and operating parameters of the Oxidor system

• primary flow vapour from the steam boiler: inlet temperature 250 °C; outlet temperature 180 °C
• second air flow from the combustion process: inlet temperature 30 °C; outlet temperature 110 °C
• max. combustion temperature in the oxidation chamber 950 °C
• holding time in the chamber 2.02 s; flow velocity ~ 7 m/sec chamber, ~ 10 m/sec outlet)
• rotary burner; fuel type: animal fat / fuel oil
• dimensions of the oxidation chamber: internal diameter – 2.8 m; length – 15.4 m; chamber volume - 95 m^3^
• max. amount of treated vapours 16,000 kg/h (~ 10,000 m^3^/h)
• fume temperature 100/105 °C
• max. amount of air from rooms 25,600 kg/h (~ 15,000 m^3^/h)
• air temperature from rooms 20/30 °C
• maximum amount of generated steam 19,500 kg/h (~ 20 t/h); steam quality - saturated; maximum operating pressure 12 bar; temperature of the supplied steam 140 °C
• 140 °C feed water temperature
• fuel consumption figures: animal fat 1317 kg/h (~ 40 GJ/h); heating oil 1193 kg/h

*The thermo-oxidator consists of the following components:*

• T01A/B rotary burner
• T01C oxidation chamber: steel structure with refractory inner casing
• T01D counter-flow diaphragm heat exchanger for preheating the air that is burnt in the thermo-oxidizer
• T01E set of supports and platform for the heat exchanger
• T01F steam boiler for the final recovery of the heat generated during the gas oxidation process
• T01G chimney
• T02A filter for cleaning the air burnt in the oxidator
• T02B/C/E valve kit for airline
• T02D centrifugal air fan, suction for air from the process
• T02F fan tubing
• T02G set of control instruments
• T03 vapour line
• T03A fan for process vapours
• T03B set of control instruments
• T03C/D/E vapour line valve kit
• T03F multi-way head for locating the required check valves in line
• T03H platform for positioning control valves
• T03I/J/K piping between cyclone and combustion chamber, combustion chamber and fan, fan and chimney
• T04 Control system: necessary automatic and manual valves and components to ensure proper process control and system safety
• T04A/B/C/D set of safety components: air fire valve, vapour fire valve, burner/chamber steam valves
• E01/E02 electrical panel and control system.

Gaseous samples were taken from the measuring points installed downstream of the exhaust fan on a vertical, steel, insulated duct with a circular cross-section and internal diameter of 1.2 m, located in front of the chimney. The measuring points were placed in accordance with the currently applicable standard for flue gas flow measurements. All given values were related to dry gas under conventional conditions (*X* ≤ 0.003 kg/kg, *p* = 1013 hPa and *T* = 273 K). The measurement results were presented in supplementary materials.

Analyses of the content of heavy metals and other elements in ashes after combustion of meat bone-meal were performed using the ICP method (Table 1). Multiple-element analyses were performed with an inductively coupled plasma spectrometer (ICP-EOS) - Philips Scientific model PU 7000, UK with an ultrasonic nebulizer (Cetac At-5000 USA).

In order to determine the parameters of the gas in the channel, the density of dry gas was determined under the contractual conditions, the static pressure in the channel, gas temperature and humidity (Table 2). These values were measured in accordance with Polish standards. The gas volume flow was measured with the EMIOTEST device. The gas velocity in the duct was determined on the basis of measuring the pressure difference with the S-type damming tube. The gas volume flow is the product of the measured gas velocity and the measurement cross-section area. Dust concentration was determined using the gravimetric method according to the standards. Two gas samples were collected for analysis by passing them through glass fibre filters placed in a dust separator with isokinetic flow control of the gas stream using a device such as EMIOTEST 2594 with heating of the filtration chamber. Samples were taken in an isokinetic manner, from representative points of the measuring cross-section of the tested emitter.

Average concentrations of inorganic compounds NO_x_, CO, CO_2_, O_2_, SO_2_ in gases from the thermo-oxidator (Table 3), were made with a portable exhaust gas analyser LANCOM Series II, a device equipped with electrochemical sensors, enabling the measurement of concentrations from 3 to 9 gases, with the possibility of measuring CO_2_ and pressure. A PHOTON microprocessor analyser equipped with an IR radiation absorption metre was also used. To determine the concentration of metals, the flue gas was passed through filters made of borosilicate glass fibres placed in a dust separator with a heated filtration chamber, and then cooled to condense the condensate. The aspiration was carried out with the control of the isokinetic flow of the intake gas stream using an EMIOTEST device.

A gas sample representative for the tested gas stream was taken in accordance with the standards. Antimony, arsenic, chromium, tin, cadmium, cobalt, manganese, copper, nickel, lead, mercury, thallium and vanadium were determined in the sample of dust retained on filters and in the condensate (Table 4). Chemical analysis was performed using the argon plasma mass detection (ICP-MS) technique.

The toxicity level of the analysed sample was expressed as the toxic equivalents (TEQ) value, which was calculated from the results of chemical analyses of the mass content of all PCDDs and PCDFs congeners having chlorine atoms in positions 2, 3, 7 and 8 (Table 6). The numerical value of TEQ is the sum of the partial values, obtained by multiplying the analytical result of the concentration of a single congener by the appropriate toxic equivalency factors (TEF).

The TEQ mass values are calculated from the Eq. (1):

(1) $$\mathrm{TEQ}=\sum_{i=17}^{i=1}\left(m_{i}\cdot \mathrm{TE}F_{i}\right)$$

where: TEQ - toxicity level of the tested sample (pg/g or ng/kg); m_i_ – mass of PCDD, PCDF_i_ -congener in (pg or ng); TEF_i_ – factor equivalent to 2,3,7,8-TCDD toxicity for the i-congener.

## CRediT Author Statement

**Zygmunt Kowalski:** Conceptualization, Methodology, Formal analysis, Investigation Conducting, Resources, Data curation, Writing- Original draft preparation, Visualization, Preparation, Writing- Reviewing and Editing; **Agnieszka Makara:** Formal analysis, Resources, Data curation, Writing- Original draft preparation, Visualization, Preparation.

## Declaration of Competing Interest

The authors declare that they have no known competing financial interests or personal relationships which have, or could be perceived to have, influenced the work reported in this article.

## Appendix Supplementary materials

Image, application 1
